# Mitochondrial epigenetic mechanisms in cancer: an updated overview

**DOI:** 10.3389/fcell.2025.1720652

**Published:** 2026-01-12

**Authors:** Andrea Stoccoro, Fabio Coppedè

**Affiliations:** Department of Translational Research and of New Surgical and Medical Technologies, University of Pisa, Pisa, Italy

**Keywords:** cancer, epigenetics, histone modifications, mitoepigenetics, mtDNA methylation, non-coding RNA

## Abstract

Mitochondria are central organelles in regulating apoptosis, cellular metabolism, metabolite biosynthesis, energy production, and overall cellular homeostasis. Over the past years, abundant evidence has shown that mitochondrial dysfunction and the resulting metabolic reprogramming profoundly influence key hallmarks of tumor development, including initiation, progression, angiogenesis, and metastasis, playing a role also in therapeutic resistance. Consequently, mitochondria have emerged as a promising target for anticancer therapy. Beyond well-known mutational abnormalities in the mitochondrial genome, recent studies indicate that altered mitochondrial epigenetic mechanisms could also contribute to cancer etiology. In the current review, we present a brief, up-to-date overview of the literature on mitochondrial epigenetic regulation in cancer. We will focus on the main characterized mitoepigenetic mechanisms, namely mitochondrial DNA (mtDNA) methylation and activity of mtDNA-encoded non-coding RNAs. We also consider bidirectional epigenetic crosstalk between the nucleus and mitochondria, whereby metabolites and signaling pathways coordinate chromatin states and mitochondrial function. Collectively, available evidence links mitoepigenetic alterations to tumor progression and pharmacoresistance, nominating these pathways as tractable targets for pharmacological intervention.

## 1 Introduction

Mitochondria are intracellular organelles that support a wide range of cellular functions, such as the ATP production and generation of reactive oxygen species (ROS), and act as key regulators of cellular homeostasis (Clemente-Suárez et al., 2023). Cancer cells are highly dependent on proper mitochondrial function, as they provide energy and molecules necessary for tumor development, including initiation, progression, therapeutic resistance, angiogenesis, and metastasis (Chen et al., 2024). During tumorigenesis, bioenergetic reprogramming shifts from the high ATP yield of oxidative phosphorylation (OXPHOS) to a metabolic state that balances energy production with the generation of substrates required for biosynthesis and rapid proliferation (Keoh et al., 2025).

Mitochondria contain their own DNA, known as mitochondrial DNA (mtDNA), which is a 16,569 bp circular molecule that encodes 22 tRNAs, 2 rRNAs, and 13 polypeptides of the electron transport chain system. Moreover, mtDNA contains some pseudogenes and encodes non-coding RNAs (ncRNAs), including long non-coding RNAs (lncRNAs) and small non-coding RNAs, such as microRNAs (miRNAs). The mtDNA contains one non-coding region, the displacement loop (D-loop), in which reside the replication and transcription start sites. Multiple nucleus-encoded proteins govern mtDNA transcription and replication, such as mitochondrial RNA polymerase and mtDNA-maintenance factor TFAM, which binds and packages mtDNA, promoting transcription initiation and genome stability (Basu et al., 2020).

Mutations in nuclear genes encoding for mitochondrial proteins, as well as in mtDNA genes, are common in cancer, but rather than disabling energy metabolism, they reconfigure mitochondrial bioenergetic and biosynthetic states (Du et al., 2025; Wallace, 2012). Recent evidence suggests that, in addition to gene mutations, epigenetic modifications may also arise during carcinogenesis. Epigenetics encompasses stable yet sequence-independent layers of gene control, including DNA methylation, histone-tail modifications, and non-coding RNA–mediated regulation, which display tissue and cell specificity and can be modulated by aging, disease states, or environmental stressors (Berdasco and Esteller, 2019). Recognition of these influences has broadened our understanding of complex diseases, including cancer, cardiovascular conditions, and neurodegenerative disorders, where epigenetic changes are being actively investigated as diagnostic and prognostic biomarkers. Importantly, the reversible nature of epigenetic marks makes them attractive targets for cancer prevention and therapy, ranging from behavioral and lifestyle-based interventions that influence epigenetic states to pharmacological agents that modulate or inhibit epigenetic enzymes (Wang K. et al., 2022). Several recent findings suggest that epigenetic mechanisms extend beyond the nuclear genome and also influence mitochondrial gene expression (Stoccoro and Coppedè, 2021). These “mitoepigenetic” mechanisms affect mtDNA transcription and replication, modulating mitochondrial biology. Here, we present a brief, up-to-date overview of the literature on mitochondrial epigenetic regulation in cancer.

## 2 Epigenetic and mitoepigenetic mechanisms

Epigenetic mechanisms are key modulators of cellular function, extensively characterized in the nuclear genome. In particular, DNA methylation, histone modifications, and ncRNAs play central roles in controlling nuclear gene expression, chromatin organization, and the three-dimensional architecture of the genome (Farsetti et al., 2023). Mitoepigenetics originally referred to epigenetic regulation of the mitochondrial genome, but is now used more broadly to describe bidirectional mito-nuclear crosstalk mediated by epigenetic mechanisms (Dong et al., 2020).

DNA methylation involves the addition of a methyl group to cytosine by DNA methyltransferases (DNMTs). In the nuclear DNA, the methylation mainly occurs at CpG sites: in promoters, it usually represses transcription, while in gene bodies, its effect is frequently associated with gene expression (Wang Q. et al., 2022). Cytosines can also undergo hydroxymethylation (5-hmC) by ten-eleven translocation (TET) enzymes, which contributes to chromatin remodeling. Although still debated, DNA methylation in mtDNA was first reported more than 40 years ago in animal models and later confirmed in human and mouse fibroblasts (Bicci et al., 2021; Mawlood et al., 2016). Renewed interest in mitoepigenetics arose when recent studies not only identified mtDNA methylation marks, but also linked them to mitochondrial regulation, through TFAM binding to methylated mtDNA and DNMT1-dependent modulation of mtDNA gene expression (Rebelo et al., 2009; Shock et al., 2011). Following research confirmed mitochondrial localization of DNMT1, DNMT3A/B, and TET enzymes, and reported correlations between mtDNA methylation, mtDNA copy number, and mtDNA gene expression (Chestnut et al., 2011; Bellizzi et al., 2012; Wong et al., 2013; Chen et al., 2012; Dzitoyeva et al., 2012). Of note, altered mitoepigenetic marks in both cellular and animal disease models were reported in several studies, as well as in human tissues of individuals affected by cardiovascular, metabolic, neurodegenerative disorders, and cancer (Stoccoro and Coppedè, 2021).

Another well-characterized epigenetic mechanism is histone tail modification. Histone proteins, organized into nucleosomes, undergo a variety of modifications that regulate chromatin accessibility and gene expression. For instance, lysine acetylation promotes gene transcription. Since mtDNA lacks histones, this epigenetic modification could not be included as a mitoepigenetic mechanism. However, mtDNA is packed into nucleoids, where the core protein is the TFAM, which exerts histone-like structural roles. Interestingly, post-translational modifications of this protein, including acetylation and phosphorylation, affect its function by modulating its affinity to mtDNA (King et al., 2018; Lu et al., 2013). TFAM post-translational modifications have been involved in various pathologies, such as sepsis-induced acute kidney injury (Deng et al., 2023), Parkinson’s disease (Wang et al., 2014), and diabetes (Santos et al., 2014). However, although TFAM expression has been involved in the etiology of several different types of cancer, until now, there is no direct evidence of a TFAM-post-translational modification role in carcinogenesis (Dong et al., 2020).

An additional epigenetic layer is mediated by ncRNAs, which exert diverse regulatory and structural functions. NcRNAs are mainly classified according to their length: the best characterized are long non-coding RNAs (>200 nucleotides), including linear (lncRNAs) and circular (circRNAs) ones, and short RNAs (<200 nucleotides), such as miRNAs and piwi-interacting RNAs (piRNAs), which are composed of about 17–23 and 27–30 nucleotides, respectively (López-Jiménez and Andrés-León, 2021). Inside the nucleus, ncRNAs interact with DNA, mRNA, and various proteins, impacting gene expression and chromatin structure. The ncRNAs are also found within mitochondria. Most of them are encoded by nuclear DNA, while a smaller fraction is encoded by the mitochondrial genome (Méndez-García et al., 2025). Alterations in the expression of mtDNA-encoded ncRNAs have been associated with several human diseases, including pulmonary hypertension (Bai et al., 2025), mood disorders (Fettahoğlu et al., 2025), Alzheimer’s disease (Xiong et al., 2024), heart failure (Yang et al., 2014) and cancer (Dong et al., 2020).

## 3 MtDNA methylation in cancer

Evidence linking mtDNA methylation to cancer remains mixed (Table 1). Early work reported little to no presence of 5-methylcytosine (5-mC) in mtDNA derived from cancer cell lines and from malignant and non-malignant tissues of patients with gastric and colorectal cancer (Maekawa et al., 2004). Similarly, low levels were detected in cervical cancer (Sun et al., 2011) and in adenomas (Morris et al., 2018).

**TABLE 1:** Mitochondrial DNA methylation and cancer.

Experimental model	Mitoepigenetic target	Observation	References
15 cancer cell lines and tissues of 31 gastric cancer and 25 colorectal cancer individuals	Methylation levels of 37 CpG sites across the mtDNA	Hypomethylation of mtDNA in all analyzed samples	Maekawa et al. (2004)
HPV16-positive exfoliated cervical lavage cells from women with cervical precancer (12 CIN2 and 27 CIN3 or squamous cell carcinoma) and from 46 women without cytological abnormalities	D-loop region methylation	mtDNA methylation was generally low (<1%), with only a few CpG sites showing disease-associated differences	Sun et al. (2011)
Tumor and corresponding non-cancerous tissues from 24 colon and 20 rectal cancer patients	D-loop region methylation	The D-loop was demethylated in most tumors compared to normal tissues. The content of the subunit of NADH, the ND2, was elevated in cancer tissues compared with the corresponding non-cancerous tissues	Feng et al. (2012)
Tumor and normal cells form 65 patients with colon cancer	D-loop region methylation	Decreased D-loop methylation and high expression of ND2 protein and mtDNA copies in cancer tissue. D-loop methylation was markedly decreased in clinicopathological stages III and IV compared with that in stages I and II.	Gao et al. (2015)
5 colorectal cancer cell lines treated with the DNA demethylating agent 5-azacytidine	D-loop region methylation	In two cancer cell lines, namely Colo-205 and lovo, 5-azacytidine induced D-loop demethylation and increased mtDNA copy number content, leading to increased cell proliferation, reduced apoptosis, and a relative cell cycle arrest in G0/G1 phase	Tong et al. (2017)
Peripheral blood from 15 women belonging to five families, each spanning three generations and including one breast cancer case	Whole mtDNA genome methylation	mtDNA methylation was conserved within families, and eight aberrant D-loop methylation sites (7 hypermethylated, 1 hypomethylated) were correlated with breast cancer	Han et al. (2017)
Normal mucosa and paired adenoma samples from 4 patients	Whole mtDNA genome methylation	Methylation levels were generally low (∼1%) in both normal and adenoma tissues, although clusters of higher methylation were detected in the ribosomal RNA genes. Methylation levels did not differ between normal and adenoma samples	Morris et al. (2018)
Glioblastoma and osteosarcoma cells at different stages of tumor progression	Whole mtDNA genome	Decreased methylation associated with tumor progression and increased mtDNA copy number. Once tumors had restored sufficient mtDNA, higher levels of 5-mC accumulated in the D-loop to restrict further replication. D-loop methylation negatively correlated with *MT-ND5* and *MT-ND6* expression	Sun et al. (2018)
Normal and cancer breast cells (MCF10A, HMEC and MCF7), and normal and cancer liver cells (hepatocytes and HepG2)	Whole mtDNA genome methylation	Methylation patterns were cell-type specific, occurring predominantly at non-CpG sites, and displaying a notable difference between normal and cancer cells	Patil et al. (2019)
Two oral squamous cell carcinoma cell lines (SAS and H103), and stem cell-like tumour spheres derived from SAS treated with cisplatin	Whole-mtDNA genome methylation	SAS tumor spheres and H103 cells exhibited lower sensitivity to cisplatin compared with SAS parental cells. Cells with reduced mtDNA content showed decreased responsiveness to the drug. Cisplatin resistance in stem cell-like spheres was not influenced by mitochondrial DNA methylation. H103 cells displayed hypermethylation of *MT-CO1* and *MT-CYB*, accompanied by increased expression of mitochondrial genes	Aminuddin et al. (2020)
Tumor and corresponding normal tissues collected from patients with liver (10 pairs) and head and neck (6 pairs) cancers	Whole-mtDNA genome methylation	Increased methylation of CpG and non-CpG sites in head and neck tumor samples compared to their matched adjacent tissues was detected. No difference in both CpG and non-CpG methylation was detected between tumor and normal liver samples	Goldsmith et al. (2021)
Fifteen sets of matched primary renal cell carcinoma (RCC) tumor tissue, tumor-adjacent healthy kidney tissue, and bone metastatic tissues	D-loop	The D-loop region in bone metastatic tumor tissues was markedly hypermethylated compared to primary RCC tumor tissues and adjacent healthy tissues. Increased D-loop methylation was associated with decreased mtDNA copy number and accumulation of DNMT1 in the mitochondria	Liu et al. (2022a)

In contrast, studies targeting the D-loop suggest disease-related hypomethylation coupled to altered gene expression. In matched colorectal specimens, the D-loop was methylated in adjacent non-cancerous tissue but unmethylated in most tumor samples, alongside higher ND2 (NADH dehydrogenase 2) protein levels in cancer tissue (Feng et al., 2012). ND2 is a subunit of Complex I (NADH dehydrogenase) of the electron transport chain, the rate-limiting complex of oxidative phosphorylation, so its increased expression may reflect enhanced Complex I activity and, consequently, elevated oxidative phosphorylation. Because cancer cells often have elevated energy demands, it can be hypothesized that D-loop demethylation promotes ND2 upregulation, ultimately contributing to increased mitochondrial energy production. Consistently, across 65 colorectal cancer specimens, the D-loop showed reduced methylation relative to paired controls, whereas the copies of mtDNA and of ND2 protein expression were upregulated (Gao et al., 2015). Notably, ND2 expression was linearly correlated with mtDNA copy number, suggesting that increased mtDNA content, together with D-loop demethylation, may contribute to ND2 upregulation as part of the enhanced energy requirements of cancer cells. Moreover, in Caco-2 and other colorectal cancer cell lines, the pharmacological demethylation induced by 5-aza-2′-deoxycytidine led to reduced D-loop methylation along with increased mtDNA copies. These changes were accompanied by altered cellular behaviors, including enhanced proliferation, reduced apoptosis, and a relative arrest in the G0/G1 phase of the cell cycle (Tong et al., 2017). Therefore, it can be hypothesized that D-loop demethylation promotes an increase in mtDNA content, likely through enhanced mtDNA replication, leading to elevated mitochondrial energy production. The resulting rise in ATP availability may support the observed cellular behaviors, including increased proliferation, reduced apoptosis, and G0/G1 cell-cycle arrest, all of which are consistent with a metabolic shift that favors tumor growth.

Extending cancer-related mtDNA methylation alterations beyond colorectal cancer, glioblastoma, and osteosarcoma models exhibited progressive declines in mtDNA methylation, accompanied by rising mtDNA copy number and *MT-ND5* and *MT-ND6* (which encode two core transmembrane subunits of Complex I) gene expression alteration. Moreover, authors observed that once mtDNA content had recovered sufficiently to initiate tumorigenesis, higher 5-mC over the D-loop emerged, potentially restraining further replication (Sun et al., 2018). These findings suggest that reduced D-loop methylation may facilitate the early expansion of mtDNA copies required to sustain tumor initiation, whereas the subsequent rise in 5-mC levels once mtDNA content is sufficiently restored may act as a regulatory brake to prevent excessive mitochondrial replication, thereby contributing to the metabolic equilibrium that supports tumorigenesis. Changes in mitochondrial DNA methylation have been implicated in breast cancer as well. In an analysis of peripheral blood from women of five families, each harboring one breast cancer case, abnormal methylation within the D-loop was associated with the presence of the disease (Han et al., 2017). Interestingly, the D-loop displayed family-specific methylation patterns, leading the authors to propose maternal inheritance of these signatures (Han et al., 2017). Although the study did not include functional assays, the authors hypothesized that altered D-loop methylation may interfere with the control of mtDNA replication and transcription, processes essential for maintaining mitochondrial copy number and oxidative metabolism, thereby potentially contributing to mitochondrial dysfunction and favoring tumorigenic transformation. A subsequent investigation examined whole-mtDNA methylation profiles in breast and liver cancer cell models compared with their normal counterparts, revealing striking differences in methylation, predominantly at non-CpG sites (Patil et al., 2019). Cancerous liver cells showed markedly higher methylation across several tRNA-encoding regions, including tRNAs for arginine and lysine, and across the D-loop, compared with primary hepatocytes. Conversely, in breast cancer cells, methylation levels across tRNA genes and the D-loop were substantially lower than in normal mammary epithelial cells. Notably, D-loop methylation inversely correlated with *MT-ND6* expression in both liver and breast contexts, further suggesting that mtDNA non-coding region methylation can modulate mitochondrial transcription. Together, these findings indicate that mtDNA methylation patterns are strongly cell-type specific, yet their functional consequence, an inverse relationship with mitochondrial gene expression, appears conserved. While functional assays were not performed, the consistent inverse relationship between D-loop methylation and *MT-ND6* expression, together with the profound cancer-specific methylation shifts, suggests that mtDNA methylation may participate in the mitochondrial reprogramming that supports tumor cell proliferation and survival. A study using oral squamous cell carcinoma cultures exhibiting variable cisplatin sensitivity showed that enhanced resistance was not attributable to overall mitochondrial DNA methylation (Aminuddin et al., 2020). However, the intrinsically cisplatin-resistant H103 line displayed three hypermethylated CpG sites within *MT-CO1* and *MT-CYB* when compared with both cisplatin-sensitive SAS cells and SAS-derived tumor spheres, which had acquired resistance through cisplatin exposure. Interestingly, these hypermethylated CpG sites correlated with slightly higher expression of the corresponding mitochondrial genes, leading the authors to speculate that gene-body methylation may enhance mitochondrial gene expression, possibly by influencing post-transcriptional processing of polycistronic mtRNAs (Aminuddin et al., 2020). Although functional validation is lacking, these observations raise the possibility that mtDNA gene-body hypermethylation in intrinsically resistant cells could subtly reprogram mitochondrial gene output and respiratory activity, thereby contributing to the metabolic adaptations that support chemoresistance in oral squamous cell carcinoma. Moreover, an increase of both CpG and non-CpG methylation of mtDNA was detected in head and neck cancer specimens when compared with non-tumor samples (Goldsmith et al., 2021). Particularly, at the single-site level, three CpG positions were significantly hypermethylated in tumors relative to matched non-tumor samples, two located within *MT-ATP6* gene and one in the short intergenic region between *MT-ND2* and *MT-CO1* genes. In the same study, no altered methylation levels were observed in liver tumor samples and the corresponding normal tissues. Although the functional impact of these changes was not directly addressed, the finding that two differentially methylated sites fall within *MT-ATP6* (Complex V) and a third lies in the intergenic region between *MT-ND2* (Complex I) and *MT-CO1* (Complex IV) suggests that tumor-associated alterations in mtDNA methylation may occur in proximity to loci involved in oxidative phosphorylation, potentially accompanying subtle mitochondrial regulatory shifts during tumorigenesis. Conversely, the absence of tumor-associated methylation changes in liver samples supports the notion that mtDNA methylation responses are tissue-specific, possibly reflecting distinct mitochondrial demands or regulatory landscapes in different tumor types. More recently, a marked increase of D-loop methylation was observed in bone metastatic tumor cells derived from primary renal cell carcinoma (RCC), compared with both the primary RCC and adjacent healthy kidney tissue. In addition, primary RCC exhibited higher D-loop methylation than healthy tissue (Liu Z. et al., 2022). Interestingly, authors correlated the high D-loop methylation levels of bone metastatic tissues with low mtDNA content and accumulation of DNMT1 inside mitochondria, suggesting that mitochondrial DNMT1 may drive hypermethylation of the D-loop in metastatic cells. Moreover, the increased D-loop methylation observed in bone-metastatic RCC cell cultures was associated with a marked decrease in the expression of multiple mtDNA-encoded OXPHOS genes, including *MT-ND2, MT-ND3, MT-ND4L, MT-ND6, MT-ATP6, MT-ATP8, MT-COI*, and *MT-COII*. *In vivo*, pharmacological demethylation with 5-Aza significantly attenuated bone metastasis formation and reduced metastatic burden. These effects were accompanied by decreased ROS and ATP production and a restoration of mitochondrial respiratory function, supporting the conclusion that D-loop-driven metabolic reprogramming is required for efficient metastatic colonization of the bone niche.

Taken together, these findings point to a model in which mtDNA methylation is neither uniformly altered nor uniformly functional across cancers, but instead reflects tumor- and tissue-specific metabolic demands. Converging evidence suggests that D-loop methylation in particular can modulate mtDNA copy number and mitochondrial transcription, thereby influencing proliferative capacity, metastatic potential, or chemoresistance. At the same time, the presence of cancer types and conditions with minimal or no mtDNA methylation changes highlights that its contribution to tumor biology is context-dependent. Overall, the emerging picture supports the view that mtDNA methylation may act as a fine-tuning layer of mitochondrial regulation in cancer, with potential diagnostic and therapeutic relevance, but requiring further mechanistic clarification.

## 4 MtDNA-encoded ncRNAs in cancer

Various ncRNAs encoded by mtDNA have been identified inside mitochondria, including various lncRNAs, circRNAs, miRNAs, and piRNAs (Sripada et al., 2012; Rackham et al., 2011; Kwon et al., 2014). In the following section, the studies that associated the expression of mtDNA-encoded ncRNA with cancer will be described (Table 2). In 2007, a non-coding RNA derived from the mitochondrial *MT-RNR2* gene, termed ncmtRNA, whose expression correlated with the replicative state of the cell, was reported (Villegas et al., 2007). Its inducibility upon mitogenic stimulation and disappearance during pharmacological cell-cycle arrest strongly link its expression to proliferative activity. Although no functional assays were performed, the authors speculate, and the expression pattern supports, that ncmtRNA may exert a pro-proliferative role. Interestingly, recently it has been reported that the ncmtRNA can enter the nucleus and associate with chromatin, functioning as a chromatin-associated RNAs (caRNAs) which form a critical layer of the epigenome that can regulate nuclear transcription (Sriram et al., 2024). Further studies are needed to identify which nuclear genes may be regulated by ncmtRNA within cancer cells. A following study revealed the presence of two mitochondrial transcripts, the ASncmtRNA-1 and ASncmtRNA-2, which are antisense transcripts derived from the same *MT-RNR2* locus that produces the sense ncmtRNA (SncmtRNA), which were downregulated in cancer cells and expressed in non-cancerous cells (Burzio et al., 2009). In normal human kidney and mouse testis, the sense SncmtRNA and ASncmtRNAs were enriched in the nucleus, particularly with heterochromatin. By contrast, in cancer cells, only SncmtRNA was expressed and associated with heterochromatin (Landerer et al., 2011). Furthermore, high expression of SncmtRNAs and downregulation of the ASncmtRNAs were found in urine cells from bladder cancer patients, and neither of them was found in control subjects (Rivas et al., 2012). Immortalization of cultured human cells with high-risk human papillomavirus (HPV-16 or 18) induced downregulation of the ASncmtRNAs and induced the expression of a new SncmtRNA, named SncmtRNA-2 (Villota et al., 2012). Particularly, transduction of cells with the HPV oncoproteins E6 and E7 was sufficient to induce the expression of SncmtRNA-2, and the knockdown of the oncogene E2 in immortalized cells re-established in a reversible manner the expression of the ASncmtRNAs. In their discussion, the authors propose that SncmtRNA-2 originates from partial processing of SncmtRNA-1, which would also release a 63-nt RNA fragment from the inverted repeat. This processing event appears to occur specifically in HPV-immortalized cells and is therefore hypothesized to contribute to the immortalization process. While the 63-nt fragment was not directly detected or functionally characterized, the authors attribute to it potential regulatory activity, possibly analogous to small regulatory RNAs involved in proliferation and transformation. By performing an *in silico* analysis, the authors noted that the predicted 63-nt fragment harbors complementarity to miR-620, which is involved in the silencing of more than 100 target mRNAs. Although entirely speculative, this model links the processing of SncmtRNA-1 into SncmtRNA-2 and the 63-nt fragment with HPV-mediated immortalization through modulation of miRNAs signalling. Of note, the same research team showed that knockdown of the ASncmtRNAs with antisense oligonucleotides (ASOs) induced cell death by apoptosis in different tumor cell lines without affecting the viability of normal cells, suggesting that the ASncmtRNAs may represent a new potential therapeutic target for cancer (Vidaurre et al., 2014). This effect appears to result from Dicer-mediated processing of the ASncmtRNA stem into microRNAs that suppress translation of “survivin” mRNA, a member of the inhibitor of apoptosis family, thereby potentiating apoptotic cell death. In a following study, the expression of the ASncmtRNA-1 and ASncmtRNA-2 was associated with apoptosis and tumor metastasis of renal cell carcinoma (Borgna et al., 2017). *In vitro* ASO-silencing of murine ASncmtRNAs triggered apoptosis in RenCa mouse renal adenocarcinoma cells but not in non-transformed murine kidney epithelial cells (Borgna et al., 2017). These mice showed decreased levels of survivin, and of proteins involved in the metastatic process, including matrix metallopeptidase 9 (MMP-9), N-cadherin and P-cadherin. In a following study, the same team developed lentiviral constructs directed against ASncmtRNAs, which triggered apoptosis in murine and human melanoma cells *in vitro* and markedly reduced tumor growth *in vivo*, further supporting the therapeutic potential of ASncmtRNA knockdown in cancer (Varas-Godoy et al., 2018). Overall, the available evidence supports a proto-oncogenic role for the sense ncmtRNA, whose expression parallels proliferative activity and is consistently elevated in tumor cells. In contrast, the antisense ASncmtRNA-1 and -2 should be tumor suppressors, as they are markedly downregulated in cancer cells. However, their complete knockdown triggers cancer-selective apoptosis, indicating that these antisense transcripts function instead as tumor-essential ncRNAs whose partial repression is a recurrent feature of transformation.

**TABLE 2:** Mitochondrial DNA-encoded ncRNA and cancer.

Experimental model	Mitoepigenetic target	Expression in cancer	Suggested functional role in cancer	Validated/proposed targets	Observation	References
Resting and proliferating cells (multiple cancer cell lines)	LncRNA (derived by *MT-RNR2*): ncmtRNA	Upregulated	Pro-proliferative	Not reported	The SncmtRNA was upregulated in proliferating cells, but not in resting cells. Induction of ncmtRNA expression stimulated cell proliferation	Villegas et al. (2007)
Various tumor and normal cell lines, and cells from various human cancer tissues, such as breast, colon, prostate, and lung tumors	LncRNA (derived by *MT-RNR2*): ASncmtRNA-1 and ASncmtRNA-2	Downregulated	Tumor-suppressor	Not reported	The expression of ASncmtRNA-1 and ASncmtRNA-2 was downregulated in tumor cell lines and in human cancer tissues, and was expressed in normal cell lines and normal human tissue	Burzio et al. (2009)
Normal and cancer cells (human and murine melanoma); human (renal) and mouse (melanoma) tumor tissues	LncRNA: SncmtRNA and the ASncmtRNA-1 and ASncmtRNA-2	SncmtRNA expressed in normal and tumor cells. ASncmtRNAs downregulated in cancer cells	Not proposed	SncmtRNA and the ASncmtRNAs associated to heterocromatin	In nuclei from normal human and mouse cells and tissues, both SncmtRNA and ASncmtRNAs were detected. In cancer cells and tissues, nuclear SncmtRNA was present, whereas ASncmtmRNAs were downregulated	Landerer et al. (2011)
Cells isolated from the urine of 24 patients with bladder cancer and from 15 healthy donors	LncRNA: SncmtRNA and the ASncmtRNA-1 and ASncmtRNA-2	SncmtRNA high expression; ASncmtRNAs low expression	Not proposed; diagnostic potential suggested	Not reported	In patients, a high expression of SncmtRNA and downregulation of the ASncmtRNAs were observed. In contrast, none of these transcripts were detected in urine samples from healthy donors	Rivas et al. (2012)
Human keratinocytes (HFK) immortalized with high risk human papillomavirus (HPV)	LncRNA: SncmtRNA-2 and ASncmtRNA-1 and ASncmtRNA-2	SncmtRNA-2 upregulated; ASncmtRNAs downregulated	Not proposed	Author proposal: SncmtRNA-2 releases a 63-nt RNA predicted to sponge miR-620, which regulates over 100 target mRNAs	Immortalization of HFK with HPV-16 or 18 causes downregulation of the ASncmtRNAs and induces the expression of the sense transcript named SncmtRNA-2	Villota et al. (2012)
Several tumor cell lines	LncRNA: ASncmtRNA-1 and ASncmtRNA-2	Downregulation	Pro-survival	ASncmtRNAs sustain survivin expression	Knockdown of the ASncmtRNAs induced cell death by apoptosis in tumor cells without affecting the viability of normal cells	Vidaurre et al. (2014)
*In vitro* (murine renal adenocarcinoma cell line RenCa cells) and *in vivo* (immunocompetent Balb/C mice inoculated with RenCa cells) models of renal cell carcinoma (RCC)	LncRNA: ASncmtRNA-1 and ASncmtRNA-2	Downregulation	Pro-survival	Knockdown of ASncmtRNAs reduces survivin and EMT-associated proteins (N-cadherin, P-cadherin, MMP-9)	*In vitro* knockdown of murine ASncmtRNAs induced apoptotic death of mouse renal adenocarcinoma RenCa cells, but not normal murine kidney epithelial cells. *In vivo* treatment provided molecular evidence for metastasis inhibition	Borgna et al. (2017)
Murine melanoma cell line B16F10 (CRL-6475), human melanoma cell line A375 (CRL-1619), and human primary epidermal neonatal melanocytes (PCS-200–012), mice with subcutaneous melanoma tumors	LncRNA: ASncmtRNA-1 and ASncmtRNA-2	Downregulation	Pro-survival	Not reported	Lentiviral constructs targeting ASncmtRNAs induced apoptosis in murine B16F10 and human A375 melanoma cells *in vitro* and significantly slowed B16F10 tumor growth *in vivo*	Varas-Godoy et al. (2018)
Various human and cell cultures. Tumour and adjacent tissue samples from 21 hepatocellular carcinoma (HCC) patients	Various circular RNAs. Focus on mecciND1 and mecciND5	Upregulated	Proto-oncogenic	Interaction with TOM40 and PNPASE to facilitate the import of cytosolic proteins into mitochondria	Hundreds of mtDNA-encoded circRNAs have been identified, some of which, including mecciND1 and mecciND5, are involved in the mitochondrial import of various proteins. The mecciND1 levels were increased in HCC tumor samples	Liu et al. (2020)
Plasma samples from 54 treatment-naïve chronic lymphocytic leukemia (CLL) patients and 40 age- and sex-matched healthy donors; CLL cell lines	4 circular RNAs. Focus on mc-COX2	Upregulated	Proto-oncogenic	Not identified	The CLL patients with high mc-COX2 levels had a significantly worse overall survival compared with CLL patients with low mc-COX2 levels. Silencing of mc-COX2 in CLL cells strengthened the anti-tumor effects of drugs when used in coordination	Wu et al. (2020)
Breast cancer cells (MCF-7, ZR-75–30, MDA-MB-231)	LncRNA: mitochondrial oxygen-responsive transcript 1 (*MTORT1*)	Downregulated	Tumor suppressor	*MTORT1* acted as sponge of miR-26a-5p leading to upregulation of its target genes, *CREB1* and *STK4*	The expression of *MTORT1* was lower in breast cancer cells compared to control cells. Knockdown of *MTORT1* enhanced cell proliferation and migration	Cheng et al. (2021)
Breast cancer cell lines (MCF-7, MDA-MB- 468 and MDA-MB-231), non-malignant breast epithelial cell line (MCF-10A), and normal and breast cancer tissue specimens	13 mitomiRs. Focus on mitomiR-5	Upregulated	Proto-oncogenic	MitomiR-5 targets the *PPARGC1A* gene	The 13 mitomiRs were differentially expressed in breast cancer cell lines, non-malignant breast epithelial cell lines, and normal and breast cancer tissue specimens	Kuthethur et al. (2022)
Breast cancer cell line MCF-7 (luminal A) and triple negative breast cancer cell lines, MDA-MB-468 (basal-like TNBC) and MDA-MB-231 (mesenchymal stem-like TNBC). Control and mouse models	13 mitomiRs. Focus on mitomiRs-3	Upregulated	Proto-oncogenic	MitomiR-3 targets *ZEB1* 3′UTR to maintain *GPX4* expression and ferroptosis resistance	Higher levels of mitomiRs in basal-like triple-negative breast cancer (TNBC) cells compared to mesenchymal stem-like TNBC cells. Inhibition of mitomiR-3 in TNBC cells promoted metabolic reprogramming toward pro-ferroptotic pathways, suppressing tumor growth *in vivo*	Kandettu et al. (2025)
40 pairs of colorectal cancer (CRC samples), including tumor samples and 40 adjacent non-tumoral samples from patients	Circular RNA: SCAR/mc-COX2	Downregulation	Tumor suppressor	Predicted ceRNA network involving 5 miRNAs and 9 mRNAs	Decrease in SCAR/mc-COX2 expression in tumor tissues compared to adjacent non-tumoral tissues. A significant relationship was observed between pathological T staging and the expression status of SCAR/mc-COX2	Nourbakhsh et al. (2025)

In breast cancer cells, it was shown that the expression of the hypoxia-induced mitochondrial lncRNA *MTORT1* (encoded from 15997 to 16569 site, spanning the *MT-TP* gene and the D-loop region) was lower compared to non-cancer cells, and that the lncRNA is involved in cellular growth and proliferation (Cheng et al., 2021). Particularly, authors proposed a tumor suppressor role for *MTORT1*, as it acted as sponge of miR-26a-5p leading to upregulation of its target genes, including the transcription factor *CREB1* and the tumor-suppressor kinase *STK4,* and inhibited breast cancer progression. In a subsequent study, thirteen previously unreported mitochondrial genome-encoded miRNAs (mitomiRs) were identified, showing differential expression across breast cancer cell lines, non-malignant breast epithelial lines, and normal and tumor breast tissues (Kuthethur et al., 2022). Of note, the authors showed that one of the 13 mitomiRs, mitomiR-5, was upregulated in tumor tissue and targeted the nuclear *PPARGC1A* gene regulating mtDNA copy number. Additionally, mitomiR-5 was more highly expressed in patients with lower-grade tumors than in those with higher-grade tumors, suggesting a link with earlier stages of breast cancer development. In a following study, the same research group showed higher levels of the 13 mitomiRs in basal-like triple-negative breast cancer (TNBC) cells compared to mesenchymal stem-like TNBC cells (Kandettu et al., 2025). Among them, 11 mitomiRs, particularly mitomiR-3, were able to bind the 3′UTR of *ZEB1*, a master regulator of epithelial-to-mesenchymal transition. Notably, inhibition of mitomiR-3 in TNBC cells promoted metabolic reprogramming toward pro-ferroptotic pathways, an iron-dependent form of regulated cell death, suppressing tumor growth in animal models.

Another non-coding RNA class with a prominent role in carcinogenesis is circRNAs, which have a stable closed-loop structure and can function as oncogenes or tumor suppressors (Szczepaniak et al., 2023). Liu et al. identified hundreds of circRNAs encoded by mitochondrial DNA across human and animal cells, including mecciND1 and mecciND*5* (derived from *MT-ND1* and *MT-ND5*, respectively), which promote the import of cytosolic proteins into mitochondria (Liu et al., 2020). They reported that mecciND1 expression, and to a lesser extent mecciND5, was increased in hepatocellular carcinoma (HCC) tumors compared with matched adjacent normal tissue. In chronic lymphocytic leukemia (CLL), several dysregulated plasma circRNAs were also identified, including four that are mitochondria-encoded (Wu et al., 2020). Among the most upregulated was hsa_circ_0089762, which derived from *MT-COX2* gene and thus named mc-COX: higher mc-COX2 levels were associated with significantly worse overall survival and *TP53* deletion, and mc-COX2 silencing in CLL cells potentiated the antitumor effects of combination therapies. More recently, the expression of the circular RNA *SCAR/mc-COX2* (has_circ_0089762) was investigated in colorectal cancer (CRC) and adjacent normal tissue samples (Nourbakhsh et al., 2025). *SCAR/mc-COX2* expression was markedly lower in tumors than in matched adjacent noncancerous tissues. Furthermore, its levels were associated with pathological T stage (depth of invasion), implicating this circRNA as a potential tumor suppressor in CRC and suggesting that its dysregulation contributes to disease pathophysiology (Nourbakhsh et al., 2025). In summary, mtDNA-encoded ncRNAs exhibit strikingly diverse and context-dependent functions across cancer types. Some transcripts, such as MTORT1 and SCAR/mc-COX2 in colorectal cancer, display tumor-suppressive behavior, whereas others, including mitomiR-5, mitomiR-3, mecciND1, and mc-COX2 in CLL, act as proto-oncogenic regulators that sustain mitochondrial biogenesis, protein import, or ferroptosis resistance. Despite these divergent phenotypes, a unifying theme emerges in which mitochondrial ncRNAs modulate key axes of mitochondrial function, ranging from mtDNA maintenance and cell-death susceptibility, thereby influencing cancer cell proliferation, metabolic adaptation, and therapy responses. These findings underscore both the functional relevance and the tissue-specific plasticity of mtDNA-encoded ncRNAs in cancer, highlighting their potential as biomarkers and therapeutic targets.

## 5 Epigenetic nuclear-mitochondrial cross-talk in cancer

A well-established, epigenetically mediated bidirectional crosstalk between the nuclear and mitochondrial genomes aligns gene expression with cellular demands and environmental cues. Multiple mechanistic routes support this communication. Retrograde signaling refers to the flow of information from mitochondria to the nucleus and occurs through ROS-, Ca^2+^- and metabolite-dependent pathways (e.g., α-ketoglutarate, succinate, fumarate, acetyl-CoA), which modulate nuclear DNA and histone modifications. Anterograde signaling, in contrast, describes the regulatory influence of nuclear-encoded factors, including DNMTs, transcription factors and ncRNAs, on mtDNA transcription, replication, methylation and mitochondrial metabolism (Shaughnessy et al., 2014). In addition, RNA-mediated communication acts in both directions: nuclear ncRNAs can localize to mitochondria and regulate mtDNA activity, whereas mitochondrial ncRNAs can translocate to the nucleus and associate with chromatin, potentially influencing transcriptional programs (Huang et al., 2021). Together, these interconnected routes establish an epigenetic continuum between the two genomes with significant implications for tumorigenesis.

For example, regarding retrograde signaling, alteration of different mitochondrial metabolites, such as succinate and isocitrate, has been linked to altered nuclear DNA methylation in gastrointestinal tumors and acute myeloid leukemia, respectively (Killian et al., 2013; Figueroa et al., 2010). Mitochondrial dysfunction can also elevate mitochondrial Ca^2+^ to activate pyruvate dehydrogenase phosphatase 1, which dephosphorylates pyruvate dehydrogenase, boosting acetyl-CoA, thus driving histone acetylation programs that promote DNA-damage repair and radioresistance in colorectal cancer (Shi et al., 2021). Furthermore, mitochondrial DNA depletion in prostate and breast cancer cells induced DNMT1 expression, leading to hypermethylation of promoters of genes involved in cancer progression, including *EDNRB*, *MGMT*, and *CDH1*, resulting in their transcriptional silencing (Xie et al., 2007).

Regarding anterograde signaling, it has been reported that expression levels of nuclear-encoded genes that influence mtDNA methylation (MTDM) are altered in HCC and breast cancer (Shi et al., 2025; Ma et al., 2023). DNA methylation of nuclear genes encoding mitochondrial proteins, particularly *POLGA,* which encodes the catalytic subunit of DNA polymerase γ that controls mtDNA replication, repair, and copy number, could also participate in the tumorigenesis of different types of cancers (Lee and St John, 2015). In addition, histone modifications following mitochondrial impairment could be important players of tumorigenesis and its pharmacoresistance (Liu Y. et al., 2022). As an example, mutations in the genes encoding isocitrate dehydrogenase 1 and 2 (*IDH1* and *IDH2*), which catalyse the conversion of isocitrate to α- KG that is involved in histone methylation dynamics, have been associated with different cancers, including gliomas (Yan et al., 2009) and acute myeloid leukaemia (Mardis et al., 2009).

Mitochondria- and nuclear-encoded ncRNAs also contribute to mitonuclear communication and may influence the pathogenesis of several cancers. As discussed in the previous section, some mtDNA-encoded ncRNAs, such as SncmtRNA and the ASncmtRNAs, are capable of translocating to the nucleus, where they associate with heterochromatin. Their altered expression across multiple tumor types (Table 2) suggests that RNA trafficking from mitochondria to the nucleus may represent an additional layer of retrograde signaling relevant to tumorigenesis. Growing evidence also indicates that lncRNAs transcribed from nuclear DNA also serve as messengers that modulate mitochondrial function in cancer cells. It has been observed that nuclear-encoded lncRNAs are key players in regulating the Warburg effect, namely the shift of cancer cells toward enhanced glycolysis and reduced OXPHOS (Yang et al., 2013). As a further example, the nuclear-encoded lncRNA MALAT1 was found to be greatly enriched in the mitochondria of hepatoma cells compared to normal cells (Zhao et al., 2020). Authors reported that MALAT1 interacted with mtDNA, including the D-loop region and *MT-COX2*, *MT-ND3*, and *MT-CYTB* genes. Notably, MALAT1 knockdown increased methylation at an mtDNA CpG site and disrupted mitochondrial transcript profiles, thereby impairing mitochondrial metabolism and altering the tumor phenotype (Zhao et al., 2020).

Moreover, several miRNAs identified inside mitochondria, and that regulate mtDNA activity, are nuclear encoded and could participate in the cancer evolution (Bandiera et al., 2011; Méndez-García et al., 2025). For example, miR-24 targets *MT-ND2* in lung carcinoma cells (Michael et al., 2017), miR-26a targets *MT-COX2* in prostate cancer (Zhang et al., 2016), miR-4485 targets *MT-RNR2* in breast cancer (Sripada et al., 2017). Overall, the nuclear-encoded miRNAs that target the mitochondrial genome have the potential to induce mitochondrial dysfunction, regulate cell proliferation and apoptosis, thus contributing to the tumorigenic process (Dong et al., 2020; Suriya Muthukumaran et al., 2022).

## 6 Conclusion and future perspectives

Mitochondrial metabolism is profoundly reprogrammed in cancer, and elucidating the mechanisms that link this reprogramming to tumorigenesis is crucial. Mitoepigenetic mechanisms are emerging as key regulators of mitochondrial function, and their alterations may contribute to cancer development. To date, studies show that mtDNA methylation is altered across multiple cancer types, is associated with tumor progression, and can contribute to pharmacoresistance (Table 1). In addition, mtDNA-encoded ncRNAs are emerging as important contributors to tumorigenesis, regulating cell proliferation and presenting potential targets for pharmacological intervention (Table 2). Furthermore, mito-nuclear epigenetic communication represents an additional regulatory axis through which mitochondrial alterations can impact nuclear gene expression and tumor progression.

**FIGURE 1:**
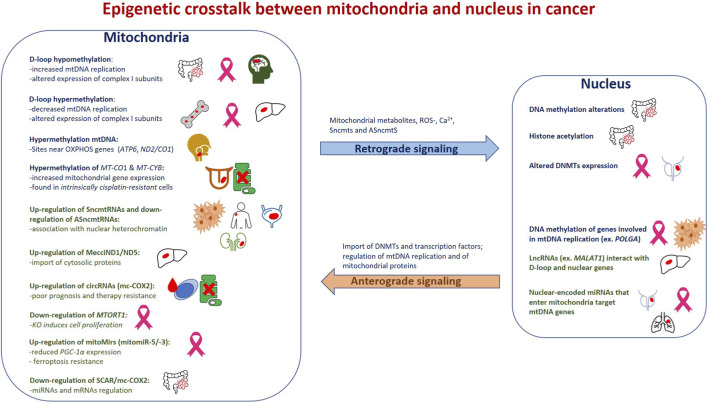
Epigenetic crosstalk between mitochondria and nucleus in cancer. Multiple mitochondrial epigenetic alterations, such as D-loop hypo/hypermethylation, gene-body mtDNA methylation, and dysregulation of mtDNA-encoded ncRNAs (SncmtRNAs, ASncmtRNAs, mitomiRs, and circRNAs) modulate mitochondrial transcription, replication, and metabolism in different types of cancer. Conversely, nuclear epigenetic processes, including DNA methylation, histone modifications, and nuclear-encoded ncRNAs, regulate mtDNA expression and mitochondrial activity. These bidirectional (retrograde and anterograde) signaling pathways contribute to tumorigenesis, metabolic remodeling, and chemoresistance.

Mitoepigenetic alterations do not act in isolation but integrate into the broader metabolic rewiring that characterizes malignant transformation. Across cancer types, changes in mtDNA methylation, the expression of mtDNA-encoded ncRNAs, and the epigenetic regulation of mito-nuclear communication collectively converge on key mitochondrial functions, such as mtDNA replication, OXPHOS activity, ROS homeostasis, metabolite production, and apoptotic signalling. Because these mitochondrial outputs feed directly into nuclear epigenetic programs, including DNA and histone modifications, mitoepigenetic mechanisms can reshape nuclear gene expression landscapes that support proliferation, metabolic plasticity, immune evasion, and metastatic dissemination. This bidirectional reinforcement between mitochondrial state and nuclear chromatin organization suggests that mitoepigenetic alterations may function as amplifiers of tumor-promoting transcriptional programs, contributing not only to tumor initiation but also to progression and therapy resistance. From a translational perspective, papers discussed in the current review indicate that mitoepigenetic changes hold promise as biomarkers of cancer detection, stratification, and treatment response. Cancer-associated patterns of D-loop methylation correlate with tumor stage (Feng et al., 2012; Gao et al., 2015), metastatic behaviour (Liu Z. et al., 2022), and, in some contexts, chemoresistance (Aminuddin et al., 2020), while several mtDNA-encoded ncRNAs show striking cancer-type specificity and remain detectable in biofluids (Table 2). Likewise, mito-nuclear epigenetic signaling driven by mitochondrial metabolites and ncRNA trafficking represents a mechanistic link between mitochondrial dysfunction and nuclear transcriptional reprogramming, underscoring its potential as a druggable axis in precision oncology. Emerging approaches aiming to modulate mtDNA methylation, inhibit oncogenic mitomiRs, restore tumor-suppressive mitochondrial ncRNAs, or interfere with metabolite-dependent retrograde signaling illustrate how mitoepigenetic pathways may be harnessed to mitigate cancer progression. In this context, the therapeutic potential of targeting ASncmtRNAs is particularly noteworthy: their repression selectively induces apoptosis in multiple tumor cell lines and markedly reduces tumor growth in animal models, underscoring their promise as mitoepigenetic tools for cancer treatment (Vidaurre et al., 2014; Borgna et al., 2017; Varas-Godoy et al., 2018). Figure 1 schematically summarizes these multilayered interactions and their potential implications for tumor biology.

Although the current review highlights the increasing interest in the field, further studies are needed to better elucidate the mitoepigenetic mechanisms underlying tumorigenesis. Future studies should investigate how mitochondrial and nuclear epigenetic programs integrate at the systems level, and how these interactions can be manipulated therapeutically. Strategies aimed at modulating mtDNA methylation, targeting mitochondria-associated ncRNAs, or exploiting metabolite-dependent epigenetic signaling pathways may offer new opportunities for cancer treatment. Further investigations should also consider the post-translational modifications of TFAM in tumorigenesis, since its expression has been reported to associate with different cancers (Dong et al., 2020). Moreover, further studies are needed to better understand the epigenetic regulation underlying the nuclear-mitochondria communication and its potential role in tumorigenesis.

Understanding these multilayered regulatory networks will be crucial for fully elucidating the role of mitoepigenetic mechanisms in cancer development, progression and therapy resistance.
